# Comparative safety and efficacy of SMAS rhytidectomy techniques: a network meta-analysis protocol

**DOI:** 10.1097/SP9.0000000000000053

**Published:** 2025-06-11

**Authors:** Grace Gasper, Sarya Swed, Yousef Tanas

**Affiliations:** a Texas A&M School of Engineering Medicine, Houston, Texas, USA; b Institute for Reconstructive Surgery, Houston Methodist Hospital, Weill Cornell Medical College, Houston, Texas, USA; c Faculty of Medicine, University of Aleppo, Aleppo, Syria

## Abstract

**Background and aim:**

Superficial musculoaponeurotic system (SMAS) rhytidectomy techniques are widely used in facelift surgery to achieve long-lasting facial rejuvenation. Nonetheless, variations in technique lead to differences in complication rates, aesthetic outcomes, and longevity of results. Current literature lacks a comprehensive network meta-analysis (NMA) that ranks these techniques based on both safety and efficacy. Thus, the aim of this study is to perform a NMA to determine their rankings based on complication and aesthetic outcomes.

**Methods and analysis:**

A network meta-analysis comparing different facelift techniques following PRISMA-NMA guidelines will be conducted. MEDLINE (PubMed), Scopus, Web of Science, Cochrane Central Register of Controlled Trials, Cochrane Database of Systematic Reviews, and Clinicaltrials.org will be searched from inception to search date. Screening, data extraction, and risk-of-bias assessments will be performed independently by two reviewers and discrepancies resolved by a third independent reviewer. Eligible studies will include randomized controlled trials (RCTs) and observational studies of adult patients undergoing a facelift procedure (SMAS plication, SMASectomy/imbrication, SMAS flap, high lateral SMAS, deep plane facelift, and composite rhytidectomy) and reporting at least one complication outcome (e.g., facial nerve injury, hematoma, seroma, skin necrosis, infection) and/or aesthetic outcome (e.g., patient satisfaction, longevity of results). R 4.4.2 Software (netmeta package) will be used to generate forest plots, treatment rankings, evaluate consistency between direct and indirect evidence, and assess heterogeneity. The ROBINS tool will be used to assess the risk of bias in nonrandomized studies and the RoB 2 tool will be used for RCTs. We will conduct a frequentist fixed- and/or random-effects NMA using the graph theory approach for each outcome. For dichotomous outcomes, odds ratios (ORs) with their corresponding 95% confidence intervals (CIs) will be calculated for all possible pairwise comparisons between the SMAS techniques. For continuous outcomes, standardized mean differences (SMDs) with 95% CIs will be calculated. Sensitivity analyses will be performed in cases of significant heterogeneity. Subgroup analyses by patient characteristics will be performed if sufficient data is available in the included studies.

## Introduction

Facelift surgery (rhytidectomy) commonly utilizes modifications of the superficial musculoaponeurotic system (SMAS) to achieve long-lasting facial rejuvenation^[1]^. SMAS rhytidectomy techniques involve manipulating the SMAS layer (a fibromuscular layer in the mid-to-lower face) to tighten and reposition sagging tissues, thereby enhancing the durability and natural appearance of a facelift^[2]^. Variations of SMAS facelift techniques include SMAS plication, SMASectomy (lateral SMASectomy)/imbrication, SMAS flap, high lateral SMAS, deep plane facelift, and composite rhytidectomy.

These techniques are well-documented in the literature^[3,4]^ and differ in their extent of dissection and tissue mobilization. Less invasive approaches (like SMAS plication or limited lateral SMASectomy) involve relatively conservative SMAS manipulation, whereas more extensive lifts (deep plane or composite rhytidectomy) release deeper retaining ligaments and mobilize a larger composite flap of tissue. The latter can yield more dramatic and potentially longer-lasting elevation of the midface, but they are also more invasive and are thought to carry higher risks (especially facial nerve injury)^[3,5]^. Conversely, simpler SMAS modifications (plication, SMASectomy) are more straightforward and have been popular due to reports of reliable results with low complication rates^[1,6]^. These variations in technique can lead to differences in patient outcomes, rendering it important to objectively analyze each approach on both safety (complication rates) and efficacy (aesthetic outcomes) merits rather than relying on subjective perceptions, particularly for efficacy.

Given the multitude of techniques and the heterogeneous data, a network meta-analysis offers a powerful solution. Traditional pairwise meta-analyses^[7,8]^ are limited in this context as they restrict comparisons to two interventions at a time and cannot leverage indirect evidence across multiple surgical approaches. Additionally, they are ill-suited to synthesize fragmented evidence from comparative studies that assess different sets of techniques. Nonetheless, an NMA allows for a simultaneous comparison of multiple treatments by combining both direct head-to-head evidence and indirect evidence across a network of studies^[9]^. In practical terms, this means we can use all available comparative data to evaluate the relative safety and efficacy of each SMAS facelift technique, even if certain techniques have never been compared directly in a single trial. The NMA framework will enable us to rank the techniques on outcomes of interest – for example, determining which approach is likely the safest overall, or which yields the most enduring aesthetic results. Such a comprehensive ranking is highly valuable for clinicians, as it provides a clear hierarchy based on the totality of evidence. To our knowledge, the literature currently lacks any published NMA analyzing both safety and efficacy outcomes of facelift techniques. We therefore propose to conduct a network meta-analysis following PRISMA-NMA guidelines to integrate all available data on SMAS plication, SMASectomy/imbrication, SMAS flap, high lateral SMAS, deep plane, and composite rhytidectomy. By evaluating both complication rates and aesthetic outcomes, this study will fill a crucial gap and inform surgeons about the comparative safety and efficacy of SMAS facelift techniques on a level playing field. The goal is to provide evidence-based guidance as to which technique (or techniques) offers a compromise between low complication risk and high, long-lasting patient satisfaction.

## Methods

### Search strategy

A comprehensive literature search will be undertaken to identify all relevant studies comparing the safety and efficacy of different SMAS rhytidectomy techniques. This search will span multiple databases, including MEDLINE (PubMed), Scopus, Web of Science, Cochrane Central Register of Controlled Trials, Cochrane Database of Systematic Reviews, and Clinicaltrials.org. The search strategy will aim to capture all relevant literature from the inception of these databases up to the date of the last search. The search terms will be carefully selected to ensure coverage of the various SMAS rhytidectomy techniques under investigation, namely SMAS plication, SMASectomy/imbrication, SMAS flap, high lateral SMAS, deep plane facelift, and composite rhytidectomy. These terms will be combined with keywords related to the outcomes of interest, which include both complications (e.g., facial nerve injury, hematoma, seroma, skin necrosis, infection)^[10]^ and aesthetic outcomes (e.g., patient satisfaction, longevity of results)^[11,12]^. The search strategy will be designed to be sensitive enough to identify all potentially relevant studies while maintaining sufficient specificity to exclude irrelevant literature^[13]^.

### Inclusion criteria

Studies will be included if they involve adult patients undergoing a facelift procedure utilizing one or more of the specified SMAS rhytidectomy techniques. Both randomized controlled trials (RCTs) and observational studies, such as cohort studies and case-control studies, will be eligible for inclusion to maximize the available evidence.^[14]^

### Exclusion criteria

Studies that do not involve the specified SMAS rhytidectomy techniques will be excluded. Case reports, Case series with fewer than 10 participants, and expert opinions will also be excluded as they do not provide comparative evidence necessary for a network meta-analysis. Studies with interventions or outcomes that are not relevant to the aim of this analysis will be excluded. Non-English articles will initially be excluded, but efforts will be made to translate eligible non-English studies where feasible.

### Study selection and data extraction

All records identified through the electronic database searches will be imported into Covidence to facilitate organization and removal of duplicate entries. The study selection process will involve two distinct stages of screening, performed independently by two reviewers. In the first stage, the titles and abstracts of all identified records will be screened against the pre-defined eligibility criteria. Any disagreements that arise between the two reviewers during this stage will be resolved through discussion and, if necessary, by consultation with a third independent reviewer to reach a consensus.

Following the initial screening of titles and abstracts, the full-text articles of all potentially eligible studies will be retrieved and subjected to a more detailed assessment. The same two independent reviewers will evaluate these full-text articles against the inclusion and exclusion criteria. Again, any discrepancies in the eligibility assessment will be resolved through discussion, with the third reviewer available to provide a final decision if consensus cannot be achieved. The entire study selection process will be documented using a PRISMA 2020 flow diagram^[15,16]^.

Data extraction will be performed independently by the same two reviewers using a standardized data extraction sheet. This sheet will be carefully designed to capture all relevant information from the included studies, including details about the study design, patient characteristics (e.g., age, gender, and any relevant comorbidities, if reported), the specific SMAS rhytidectomy technique(s) employed in each treatment arm, the definition and timing of all reported complication outcomes, the measures used to assess aesthetic outcomes (e.g., patient satisfaction scales, objective measures of facial rejuvenation, and the timing of these assessments), and the duration of follow-up. To ensure the sheet is comprehensive and consistently applied, it will be pilot tested on a subset of the included studies prior to the commencement of full data extraction. Following independent data extraction by both reviewers, the extracted data will be cross-checked for accuracy and completeness. Any discrepancies identified during this process will be resolved through discussion between the two reviewers. If a consensus cannot be reached, the third reviewer will be consulted to arbitrate and ensure the accuracy of the extracted data.

### Risk of bias assessment

The choice of the appropriate risk of bias assessment tool will depend on the study design^[17]^. For all non-randomized observational studies that meet the inclusion criteria, the Risk Of Bias In Non-randomized Studies-of Interventions (ROBINS-I) tool will be employed. For any randomized controlled trials (RCTs) included in the analysis, the Cochrane Risk of Bias tool 2.0 (RoB 2) will be used to assess the risk of bias^[18]^. Similar to the data extraction process, the risk of bias assessment for each included study will be performed independently by two reviewers. Any disagreements in the risk of bias judgments between the two reviewers will be resolved through discussion. If necessary, the third reviewer will be involved to reach a final consensus on the risk of bias for each study. The results of the risk of bias assessment for each individual study will be summarized and will play a crucial role in the interpretation of the findings from the network meta-analysis, particularly during sensitivity analyses where the influence of studies with a high risk of bias on the overall results will be examined.

### Data synthesis and statistical analysis

The statistical analysis for this network meta-analysis will be conducted using R version 4.4.2 Software, leveraging the capabilities of the “netmeta” package^[19]^. A network meta-analysis will be performed to simultaneously compare the different SMAS rhytidectomy techniques for each of the identified complication and aesthetic outcomes. Both frequentist fixed-effects and random-effects models will be fitted using the graph theory approach, as implemented in the “netmeta” package. The choice between the fixed-effects model (assuming a common treatment effect across all studies) and the random-effects model (allowing for heterogeneity in treatment effects between studies) will be guided by the assessment of heterogeneity.

For dichotomous outcomes, such as the presence or absence of specific complications, odds ratios (ORs) with their corresponding 95% confidence intervals (CIs) will be estimated for all possible pairwise comparisons between the SMAS techniques. For continuous outcomes, such as patient satisfaction scores (if reported on a consistent scale) or measures of longevity (if quantifiable), standardized mean differences (SMDs) with 95% CIs will be calculated if the outcome is measured using the same method across studies.

To visualize the available evidence, network diagrams will be generated. These diagrams will depict the different SMAS rhytidectomy techniques as nodes, and the direct comparisons between them (as reported in the included studies) as edges. The thickness of the edges may be proportional to the number of studies or the number of participants contributing to each direct comparison. Forest plots will be generated to display the results of the network meta-analysis for each outcome. These plots will show the estimated effect sizes (ORs or SMDs) and their 95% CIs for all pairwise comparisons of the SMAS techniques. Treatment rankings will be determined based on the surface under the cumulative ranking (SUCRA) curve or by calculating the probability of each treatment being the best for each outcome.

Heterogeneity will be statistically assessed for each direct comparison and for the overall network using Cochran’s *Q* test and the *I*^2^ statistic. The *I*^2^ statistic quantifies the percentage of variation across studies that is due to heterogeneity rather than chance. Significant heterogeneity (*I*^2^> 50%) will be further explored through pre-planned sensitivity analyses. Consistence between direct and indirect evidence within the network will be evaluated using appropriate methods available in the “netmeta” package, such as the loop-specific approach or the global inconsistency test. Inconsistency can indicate potential biases or violations of the transitivity assumption, which is a fundamental requirement for the validity of indirect comparisons in network meta-analysis. We will assess the assumption of transitivity by evaluating the comparability of studies in terms of patient demographics, baseline facial aging severity, surgical expertise, and follow-up duration, which are potential effect modifiers across comparisons.

Publication bias and small-study effects will be explored using comparison-adjusted funnel plots. In addition, Egger’s regression test will be applied where appropriate, depending on the number of studies per comparison.

### Sensitivity and subgroup analyses

Sensitivity analyses may include excluding studies that were assessed as having a high risk of bias based on the ROBINS-I and RoB 2 tools; comparing the results obtained from the fixed-effects and random-effects models; and excluding studies with specific characteristics, such as those with a very short duration of follow-up, to evaluate whether they unduly influence the overall conclusions.

Subgroup analyses will be performed if there is sufficient data available in the included studies to explore potential variations in outcomes based on specific patient characteristics. These characteristics may include age, gender, or the baseline severity of facial aging, provided that these factors are consistently reported across a sufficient number of studies. The specific subgroups to be analyzed will be determined based on the patient characteristics that are most frequently and consistently reported in the included literature. These analyses will help to determine if the comparative safety and efficacy of different SMAS techniques vary across different patient populations.

## Discussion

The aim of this network meta-analysis is to provide an objective evaluation of the different SMAS rhytidectomy techniques based on both their safety profiles (as indicated by complication rates) and their efficacy (as indicated by aesthetic outcomes, longevity of results, and patient satisfaction). This will include both direct head-to-head comparisons and indirect comparisons synthesized through the network meta-analysis framework^[20]^. These findings are expected to have significant clinical implications. By providing an evidence-based ranking, this study will potentially guide surgical decision-making, allowing surgeons to select the technique that offers the most favorable balance between safety and efficacy for their patients. For instance, the analysis may identify techniques associated with a lower risk of specific complications, such as facial nerve injury or hematoma, or those that are more likely to result in higher patient satisfaction or longer-lasting aesthetic improvements. Furthermore, the quantitative estimates of the relative risks and benefits of each technique compared to others will offer surgeons more precise information to use during patient counseling, enabling them to have more informed discussions about the expected outcomes and potential risks associated with different surgical approaches.

Although there are previously published systematic reviews attempting to compare different SMAS techniques, they have significant limitations that do not allow for drawing definitive conclusions^[14]^. A recent systematic review by Mortada *et al*^[1]^ explored the evolution of SMAS facelift techniques, focusing on complications and outcomes. Their review included 27 studies and had only descriptive statistics summarizing the complication rates reported in their included studies without performing any form of comparative analysis (e.g., head-to-head pairwise or network meta-analysis), rendering it difficult to objectively compare the techniques. Our study will build upon this work by employing network meta-analysis to provide a comparative ranking of these techniques.

Another meta-analysis published in 2019 by Jacono *et al*^[3]^ specifically investigated only complication rates among different SMAS facelift techniques, offering a direct point of comparison for the safety outcomes assessed in our analysis. Although this review included 183 studies and a comparative analysis was performed, aesthetic outcomes, longevity of results, and patient satisfaction were not assessed. The study lacked any assessments of the risk of bias and did not sufficiently describe their methods of analysis. Further, there have been many SMAS rhytidectomy primary studies published after 2019 that could significantly alter their findings and therefore an updated meta-analysis is needed.

Additionally, the review by Meretsky *et al*^[21]^ aimed to compare different SMAS techniques and their complication rates. Nonetheless, their initial search of PubMed yielded an astoundingly low number of studies – 15 – and only 13 studies were included in their final review. This may have led to significant selection bias considering the fact that their study was published in 2024 – 5 years after the study by Jacono *et al*^[3]^ which included 183 studies. In addition, no comparative analysis was performed to allow for objective comparisons.

Further, the relative performance of SMAS techniques may vary across patient profiles. For instance, younger patients with mild laxity may benefit from less invasive options like SMAS plication, whereas older patients with advanced ptosis may require deeper plane techniques. Where data permits, subgroup analyses will aim to clarify these individualized recommendations. Therefore, by ensuring a comprehensive database search and integrating both direct and indirect evidence, our network meta-analysis will address the limitations of these earlier reviews, which often relied on pairwise comparisons and may not have provided a comprehensive simultaneous comparison of all relevant techniques^[22]^. Any consistencies or discrepancies between the findings of our NMA and the existing literature will be thoroughly discussed, highlighting areas of agreement or ongoing debate within the field of facial plastic surgery.

This study also highlights the inherent challenge in analyzing techniques with fragmented evidence and a high reliance on observational data. Future research should consider integrating artificial intelligence to model complex multi-dimensional outcomes. Graph Neural Networks (GNNs), for example, can represent “technique-outcome” relationships in network structures, helping overcome limitations of sparse comparative data. Furthermore, clinical imaging combined with computer vision may enable more objective assessments of facial rejuvenation than satisfaction scores alone. Models capable of balancing complication risk with aesthetic benefit could support tailored surgical planning. The evolving role of AI in surgical decision-making deserves dedicated exploration^[23,24]^.

### Potential strengths and limitations

This network meta-analysis will possess several notable strengths that mitigate the limitations of prior reviews. The comprehensive search strategy across at least five major databases aims to identify all relevant published studies, minimizing the risk of missing pertinent evidence. A key strength lies in the simultaneous comparison of multiple surgical techniques within a single analytical framework, allowing for the synthesis of both direct and indirect evidence to generate more precise estimates of relative safety and efficacy. Further, the assessment of the risk of bias in the included non-randomized studies using the ROBINS-I tool, along with the use of RoB 2 for RCTs, will provide a thorough evaluation of the quality of evidence.

Despite these strengths, several potential limitations should be acknowledged. The included studies may potentially exhibit heterogeneity in terms of patient populations, the precise execution of surgical techniques, the definitions and measurement of outcomes, and the duration of follow-up. Aesthetic outcomes can be reported in a heterogeneous manner, potentially involving subjective assessments and varying measurement scales. This heterogeneity could potentially impact the validity of the pooled estimates. The validity of indirect comparisons in network meta-analysis relies on the assumption of transitivity, which implies that the relative treatment effects are consistent across different sets of studies. Violations of this assumption could lead to biased results. While the inclusion of observational studies broadens the evidence base, these studies are inherently more susceptible to confounding and other biases compared to RCTs, a factor that will be addressed through the risk of bias assessment and sensitivity analyses. Finally, despite a comprehensive search strategy, the possibility of publication bias, where studies with statistically significant or positive results are more likely to be published, cannot be entirely ruled out – a common limitation with most meta-analyses.

Nonetheless, the findings of this network meta-analysis have the potential to significantly impact surgical practice by offering a more evidence-based understanding of the comparative safety and efficacy of various SMAS rhytidectomy techniques. The identification of techniques that balance between efficacy and high safety profile will help inform clinical decision-making, allowing surgeons to tailor their approach to individual patient needs and preferences. This could lead to improved patient outcomes and satisfaction. Moreover, the quantitative estimates of the relative risks and benefits associated with each technique can enhance the quality and objectivity of patient counseling, enabling surgeons to provide more accurate and comprehensive information about the expected results and potential complications.

## Data Availability

Datasets generated during and/or analyzed during the current study are publicly available.

## Research registration unique identifying number (UIN)

Systematic review registration: PROSPERO CRD420251012673
